# Gaseous Enemata

**Published:** 1887-08

**Authors:** J. Solis Cohen


### GASEOUS ENEMATA.

By J. Solis Cohen, M. D.,

(A Lecture delivered before the Philadelphia County Medical Society in the German Hospital, Philadelphia, March 30th, 1887.)

It is now some four years, gentlemen, since Dr. Bergeon commenced a series of experiments by the injection of hydrogen sulphide and carbon dioxide or the carbonic acid of the old nomenclature, as a therapeutic means in consumption.

On the 12th of July of last year the doctor introduced the matter to the Academy of Sciences, and on August 20th to the Congress of the French Association for the Advancement of Sciences, and on the 19th of October Prof. Cernil read a paper before the Academy of Medicine at Paris.

The beneficial results claimed for the treatment have been confirmed by a number of French physicians and by Dr. Bennett, an Englishman as you know, practising in France.

A letter from Dr. Bennett, in the *British Medical Journal*, so strongly impressed me that I wrote to Paris at once and obtained an appaıatus as devised by Morel, a pupil of Bergeon, having previously received his pamphlet, from which I have learned much that I have to communicate, most of it in his own phraseology. After its arrival Mr. Kiner, of the Polyclinic, imitated the apparatus, which can be procured for ten dollars.

While unprepared at this early date to make a decided statement as to its powers I do not hesitate to express my opinion of its value as a therapeutic measure, and I have, therefore, invited you here to see the process. The process is simple, but requires some precautions in its management to insure the success of the operation.

The noxious results of consumption are due to a septicæmia, produced by the absorption of the purulent products formed in the affected parts. A prolonged bath of the surfaces so affected with an antiseptic agent would prevent the accumulation of these matters and thus prevent the occurrence of septicæmic condition, and so far conduce to the recovery of the patient.

Hydrogen sulphide is such an agent; but when inhaled in sufficient quantity it is poisonous to the individual and cannot, therefore, be administered by inhalation, and the same may be said of other agents.

Dr. Bergeon, reasoning on some experiments of Claude Bernard, has found that certain antiseptic agents can be administered by the rectum in sufficient quantity without causing any deleterious effects to the patient, provided it is done slowly and not in too great quantity.

Claude Bernard demonstrated that gases which were introduced into the rectum could not reach the arterial system, because they must pass through the portal system and right side of the heart, and were thrown out by the lungs.

Bernard injected hydrogen sulphide into the veins of a dog, and also into the rectum, and always found the gas soon present in the exhalations of the lungs. Hence he came to the conclusion that this substance could be introduced into the rectum with impunity. Bergeon first experimented on dogs with chlorine, turpentine, ammonia, ether, bromine, etc., but these produced points of sphacelus in the mucous membrane, inflammation of the rectum in some instances and had to be abandoned. Afterwards he used carbon dioxide with hydrogen sulphide. The carbonic acid gas acts as a diluent of the sulphide, and probably has also an anæsthetic effect upon the mucous membranes as it is absorbed by the intestines, thus preventing any pain which might be caused by the sulphuretted hydrogen. Where other liquids are used the anæsthetic effect of carbonic acid gas may have some effect in subduing their hurtful effects.

Some of you may remember how the late Dr. Demme used to show the anæsthetic effects of carbonic acid gas. He used to put his arm in a bell glass filled with carbonic acid, and then let us strike the arm with a lancet: the blow of the lancet would not be felt.

Now in this operation it is necessary first to produce a pure gas, and then to pass it through a medicated liquid. The apparatus consists of two parts: one for the evolution and storing of the carbon dioxide, the other for the passage of this gas through the medicated water. The first part of the apparatus consists of a bottle for generator, furnished with a funnel tube and a delivery tube, which is attached to a rubber bag for the storage of the gas.

In this French apparatus a square bottle has been used, probably because it affords a greater surface for action to take place. The second part of the apparatus is a rubber bulb, with which to pump the gas out of the bag and through the liquid containing the sulphuretted hydrogen; attached to the bulb by a tube is a metal connection from which a rubber tube passes to the bottom of this reservoir; at the end of this tube is a valve opening outward, thus allowing the gas to pass out, but preventing its return by the same passage. After bubbling up through the liquid in this bottle the gas passes out of a minute opening near the top of the tube into the tube which conveys it to the rectum. Instead of the French apparatus we use also a modification of the ordinary Woulff bottle, which we think better as it gives freer exit to the gas. To produce the carbon dioxide gas some bicarbonate of sodium is placed in this bottle or generator, and then a mixture of sulphuric acid and water is allowed to flow slowly down the funnel tube into the bottle. The proportion of this acid mixture is one to four by weight, that is by measure 1 drachm of sulphuric acid to 1 ounce of water. Of course the moment the acid is allowed to fall upon the bicarbonate of sodium the gas begins to be generated. We allow a little of this to escape in order to drive out the air from the bag as we roll the bag up tightly.

This is one of the important points you have to attend to, because the injection of air into the intestines might cause pain.

Then connection is made with the generator, and the bag is allowed to be filled with the gas. After the bag is filled, then comes the operation of injection. This is done in this manner:

before the rectal tube is inserted, gas pumped through the liquid in the second bottle for a short time by means of the rubber bulb, so as to insure the driving out of all air from the apparatus, after which the rectal tube is inserted, and the gas slowly injected.

Dr. Bergeon, after trying all kinds of waters, came to the conclusion that the natural sulphuretted waters were best. He used the waters of Eaux Bonnes. We use the red sulphur water, which is furnished by Mr. Shafer at $4.80 by the case of twenty-four bottles. One bottle is generally used for two injections, but recently we have had a water from Michigan from Mt. Clements, which answers for three or four injections. Here is a sample of this water, which I will pass round. This has been used three times, and is still strong enough of the sulphur to do for one more injection.

Now, you see, as the pumping is done, the gas bubbles up through the liquid and mixes with it, and is passed out through the second tube into the rectal tube. The only modification which I have made is to place the bottle in hot water, or put in some warm water; and, on looking over some French journals, I find the French physicians sometimes put a little hot water in the bottle too.

Here is one of our patients who is under this treatment, and here is his temperature sheet. You see his temperature has come down from a 102° to 90°. The man is very much better than at the time treatment began; his cough is less, his expectoration has diminished, and his appetite is very much better. All the patients have not done as well as this man.

In making these injections it does not make any difference whether the patient's bowels have been moved or not, or whether the patient is undergoing menstruation. They should not be given after a full meal; the best time is three hours after breakfast and three hours after supper. They may be taken before meals. It is well to have a bed-pan near, for the injection sometimes produces a disposition to stool.

You place your hand upon the abdomen of the patient while the injection is going on, and if you find that the colon is much distended by the gas, or that there is a little pain, which sometimes arises during the operation, you then wait until the pain or distention is gone, and then go on with the injection. In from four to five minutes, sometimes in less than a minute, you can perceive the smell of the sulphurous gas in the breath.

This injection must be done slowly: it takes about fifteen to twenty minutes; absorption of the gas consuming half an hour; sometimes three-quarters of an hour. When, in cases of much impaired respiration, elimination of the gas takes place slowly, the injections must be done slowly also.

All the publications of this treatment give as a result a marked improvement in all the symptoms. Some of his more than two hundred patients Dr. Bergeon believes cured; some of them have been able to resume laborious occupations. Some consider themselves cured at the end of a few weeks and stop the treatment, with the result that a return of their bad symptoms takes place; it is therefore important that treatment should be continued for some months.

Treatment should also be renewed, from time to time, even after the disappearance of all the symptoms. Not only pulmonary tuberculosis is said to be cured, but the pharyngeal and laryngeal ulcerations also. Some of my own patients have done verv well, and one of my patients insists that she is well, but she is not. Her expectoration has merely ceased and her cough diminished in frequency. Hope of recovery has much to do with it. I underwent the some experience with the inhalations of oxygen some years ago.

Try it, gentlemen; try this new method, and Philadelphia will soon be able to say what value is to be attached to it as a therapeutic measure. In any instance your patients will not have been injured.

Should it be desired to introduce other substances into the injection, as iodoform, sulphide of carbon, etc., it may be done by placing the substance between absorbent cotton in a glass tube, which is then inserted between the second bottle and the rectal tube, so that the gas, as it comes from the sulphur water, must pass through it.

In addition to pulmonary phthisis, the following diseases are said to be cured or benefited by these injections: asthma, whooping-cough, bronchitis, pulmonary catarrh, typhoid fever, puerperal fever, and septicæmia.

In some extracts from a letter of Dr. Bennett's he reports that in two or three weeks there is generally amelioration of the symptoms, and in a few months the expectoration loses its purulent character. It takes from fifteen to twenty months to perfect a cure.

I believe they have been using this method of late at Blockley, and with good results. One patient at the Polyclinic did well for a few days, and then he became worse, had a bad taste in his mouth and a pain in his stomach, and refused to allow it to be used any more. Then we used chlorine, about 1 part to 8,000, and then his temperature went down and he began to improve again. We did not induce any irritation of the rectum by the use of the chlorine, but this may have been due to its being so weak a mixture of chlorine.—*Medical Reporter*.

